# A randomised control crossover trial of a theory based intervention to improve sun-safe and healthy behaviours in construction workers: study protocol

**DOI:** 10.1186/s12889-018-5164-8

**Published:** 2018-02-15

**Authors:** Amanda Nioi, Charlotte Wendelboe-Nelson, Sue Cowan, Hilary Cowie, Shahzad Rashid, Peter Ritchie, Mark Cherrie, Terry C. Lansdown, John W. Cherrie

**Affiliations:** 1 Heriot-Watt University, School of Engineering and Physical Sciences, Institute of Biological Chemistry, Biophysics and Bioengineering, Edinburgh, EH14 4AS UK; 2 Heriot Watt University, School of Social Sciences, Department of Psychology, Edinburgh, EH14 4AS UK; 3 Institute of Occupational Medicine, Research Avenue North, Edinburgh, EH14 4AP UK; 4 University of Edinburgh, Centre for Research on Environment, Society and Health, School of Geosciences, Edinburgh, EH8 9XP UK

**Keywords:** Skin cancer, Vitamin D, Theory of planned behaviour, Sun-safety, Healthy behaviours, Text messaging intervention

## Abstract

**Background:**

Exposure to sunlight can have both positive and negative health impacts. Excessive exposure to ultra-violet (UV) radiation from the sun can cause skin cancer, however insufficient exposure to sunlight has a detrimental effect on production of Vitamin D. In the construction industry there are onsite proactive behaviours for safety, but sun-safety remains a low priority. There is limited research on understanding the barriers to adopting sun-safe behaviours and the association this may have with Vitamin D production. This paper reports a protocol for an intervention study, using text messaging in combination with a supportive smartphone App. The intervention aims to both reduce UV exposure during months with higher UV levels and promote appropriate dietary changes to boost Vitamin D levels during months with low UV levels.

**Method/design:**

Approximately 60 construction workers will be recruited across the United Kingdom. A randomised control crossover trial (RCCT) will be used to test the intervention, with randomisation at site level – i.e. participants will receive both the control (no text messages or supportive App support) and intervention (daily text messages and supportive App). Using the Theory of Planned Behaviour (TPB) the intervention focuses on supporting sun-safety and healthy dietary decisions in relation to Vitamin D intake. The intervention emphasises cultivating the perception of normative support in the workplace, increasing awareness of control and self-efficacy in taking sun-protective behaviours, making healthier eating choices to boost Vitamin D, and tackling stigmas attached to image and group norms. Each study epoch will last 21 days with intervention text messages delivered on workdays only. The supportive App will provide supplementary information about sun protective behaviours and healthy dietary choices. The primary outcome measure is 25-hydroxy-Vitamin D [25(OH)D] level (obtained using blood spot sampling), which will be taken pre and post control and intervention periods. Secondary outcome measures are two-fold, (1) using the TPB to detect changes in behaviour, and (2) quantifying UV exposure during the UK peak radiation season (April–September) using body-mounted UV sensors.

**Discussion:**

This study will provide important information about the effectiveness of a technology-based intervention to promote sun-safety and healthy behaviours in outdoor construction workers.

**Trial registration:**

ISRCTN15888934* retrospectively registered* 15.01.2018.

**Electronic supplementary material:**

The online version of this article (10.1186/s12889-018-5164-8) contains supplementary material, which is available to authorized users.

## Background

Exposure to sunlight can have both positive and negative health impacts. Excessive exposure to ultra-violet (UV) radiation from the sun can cause skin cancer and in Britain it has been estimated that each year there are almost 3000 cases of non-melanoma skin cancer and 250 cases of malignant melanoma, caused by exposure to UV from sun at work [1–3]. However, insufficient exposure to sunlight has a detrimental effect on the production of 25(OH)D, which may result in bone pain and osteoporosis. Low 25(OH)D has also been associated with increases in the risk of some cancers, cardiovascular diseases, metabolic disorders, infectious diseases, and autoimmune diseases; although the evidence for causal associations is mostly still equivocal [4]. Vitamin D insufficiency (defined as 11-20 ng/mL concentration of 25(OH)D, [5]) may increase the risk of elevated blood pressure and vascular events, both heart attacks and stroke [6]. However, it is unclear whether these effects are due to circulating levels of 25(OH)D or to exposure to sunlight (UVA-induced production of Nitric acid which has vasodilating effects and UVB involved in Vitamin D production), [7, 8]. A recent European study suggested that blood 25(OH)D levels are often below recommended ranges (Vitamin D deficiency is often defined as plasma 25(OH)D < 25 nmol/l) for the general population, and that consumption of foods rich in Vitamin D (naturally rich or fortified) and use of Vitamin D supplements is low [9]. However, in the United Kingdom (UK) a Vitamin D deficiency is defined as < 30 nmol/l [10]. Official advice from the UK National Health Service (NHS) is now that all adults should consider taking a daily supplement containing 10mcg of Vitamin D during the autumn and winter months.

The issue of communicating sun-safety is complicated in Britain. Although individuals are typically aware of the dangers of too much sun exposure in the summer, there is a large part of the year when people are unlikely to get sufficient UV exposure to synthesise the Vitamin D necessary for health [11]. For example, from around October to April in southern England the Ultra Violet Index (UVI) level (a categorical UV scale developed by the World Health Organisation for risk communication: ranging from 1 to 11+) is less than 2 [12]. Such levels are considered sufficiently low not to require sun protection measures and consequently there is minimal Vitamin D synthesis [13]. The main source of Vitamin D during these periods is diet, with foods such as fish oils and fatty fish high in Vitamin D (over 500 IU per serving - equivalent to 50 μ-g Vitamin D), and beef livers, cheese and egg yolks also containing lower quantities (less than 50 IU per serving). Fortified cereals and irradiated mushrooms can also provide moderate levels of Vitamin D, although this varies by manufacturer and species, respectively (U.S. Department of Health & Human Services, [14]). In essence, the message for sun-safety needs to be tailored to the outdoor conditions (not simply determined by UV Index) in a way that is impossible to deliver in a single intervention [15]. Use of a variable set of messages to promote sun-safety and healthy eating behaviour, presented via smartphone, may be an attractive approach to delivering a variable and complex health promotion intervention [16].

Short messaging services (SMS) have been shown to be effective in health promotion intervention studies [17, 18]. A recent systematic review has also shown that interventions using SMS or multi-media messages generally produce statistically significant positive behavioural change [19]. One small study (conducted September to December, 2005) designed to increase physical activity using messages delivered via the Internet and mobile phone technology showed increases in moderate activity and increased loss of body fat in the test group [20]. The role of both positive and negative messages, along with the influence of personal beliefs and risks, and the attitudes of others towards the intended behaviour change may be important factors in successful workplace interventions for UV exposure.

Awareness of the risk of excessive UV exposure and skin cancer is high in the UK. The message of associations between UV and Vitamin D production remains ambiguous [21]. However, there is evidence to suggest that awareness of the association between UV and Vitamin D is increasing due to the number of people requesting 25(OH)D tests [22]. There are existing studies that target rising awareness of skin cancer risks [23–25]. Research investigating the underlying psychological constructs as to why people may or may not perform sun-safety or healthy eating behaviours that enhance Vitamin D levels are more limited. Research has indicated that behavioural choices involve the interrelation of social factors and an individual’s cognitions [26]. These can include normative social influences - attitudes, self-efficacy respectively [27]. Further, for those in the construction industries there are likely to be barriers to performance of sun-safety behaviours. For example, practical difficulties of covering up or wearing sun screen, along with the attitudes of fellow workers, employers and regulators [28]. It is probable that changing behaviour will require a more sustained communication programme, designed to target specifically the multiplicity of factors that determine the relevant behaviour(s) in the population of interest.

**Fig. 1:**
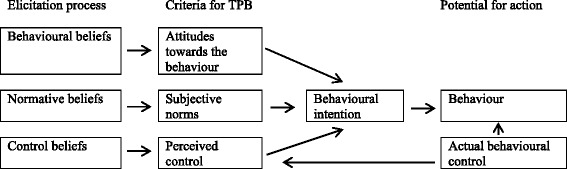
Theory of Planned Behaviour framework (Ajzen and Fishbein, 1980)

The Theory of Planned Behaviour (TPB) [29, 30] is a well-established theoretical model, which has been used to investigate decision-making processes by attempting to understand attitude-behaviour interactions (Fig. 1). The theory classifies ‘behavioural intention’ as the determinant of ‘behavioural change’. According to the TPB, behaviour is influenced by three factors: i) behavioural beliefs (beliefs about the likely outcomes of the behaviour, and the evaluations of these outcomes); ii) normative beliefs (beliefs about the normative expectations of significant others, and motivation to comply with these expectations); and iii) control beliefs (beliefs about the existence of factors that might facilitate or constrain performance of the behaviour, and the power of these factors). Behavioural beliefs produce a favourable or unfavourable attitude towards the behaviour; normative beliefs result in perceived social pressure to perform the behaviour (subjective norms); and control beliefs result in perceived behavioural control. Behavioural intention, the proximal determinant of behaviour, is predicted by the outcome of the attitude, subjective norm, and perceived behavioural control. An understanding of the various psychological constructs that guide particular behaviours is important if interventions are to be designed to effect behavioural change [29].

There are also other factors that might influence performance of the desired behaviours. For example, for sun-safety and healthy eating behaviours (to increase in Vitamin D levels), knowledge of the subject under investigation (i.e. sun-safety and the links with Vitamin D synthesis) may be pertinent to the performance of positive behaviour. Knowledge may not directly effect change in behaviour but more likely help identify underlying beliefs around sun-safety or beliefs that might exist around Vitamin D synthesis given the limited public information available [31].

In addition to developing an understanding of attitudes and beliefs, it is helpful to understand the populations’ readiness to make behavioural changes. One approach to address this is the Stages of Change model [32]. Constructs within the model represent temporal changes and readiness to act. There are five stages: (i) pre-contemplation (not ready) – no action is intended to be taken; (ii) contemplation (getting ready) – people intend to change in the next 6 months; (iii) preparation (ready) - people intend to act in the immediate future; (iv) action - people have made specific modifications to their lifestyle within the last 6 months; and (v) maintenance – people have made overt modifications to their lifestyle and intend to continue maintaining changes.

### Formative work

Implementation of the TPB requires some prior formative work to determine the beliefs most commonly held by the target study population. In this case, outdoor construction workers’ beliefs about sun-safety, and healthy eating in relation to increasing Vitamin D levels were elicited through their participation in focus groups and using postal questionnaires.

Two focus groups were held in central Scotland with five participants in each and lasting approximately 1.5 h. Data were audio-recorded and subsequently transcribed. Due to the complex, and time-consuming logistics of setting up focus groups, it was decided to use postal questionnaires instead. Both methods of elicitation are commonly used in TPB studies, and the choice of which to use is a pragmatic one. Questionnaires were distributed to 94 construction workers in both Scotland and England. Sixty-six completed questionnaires (70%) were returned.

Participants who took part in focus groups, and who completed questionnaires were asked the same questions. The elicitation questions were designed to identify (i) the most commonly held beliefs in relation to the advantages and disadvantages of performing the target behaviours; (ii) the most important individuals or groups of people who would approve or disapprove of performing the target behaviours; and (iii) factors perceived to facilitate or constrain performance of the target behaviours. Half of the participants who received the questionnaire answered questions on sun-safety first and then healthy eating to increase Vitamin D, and the other half answered in the reverse order.

The most commonly held beliefs were then used to construct a draft questionnaire, which comprised a series of seven point Likert-type items designed to measure all of the TPB theoretical constructs (see Fig. 1 above). This draft was piloted on a further 46 construction workers.

Item-total correlations were calculated for those sets of items comprising measures of behavioural intention, attitude, subjective norm and perceived behavioural control. To ensure a high degree of internal consistency of each of the measures, any item with an item-total correlation of less than 0.3 was dropped from the final questionnaire.

### Pilot study

The authors piloted the intervention in operatives working at a construction site in central Scotland (*n* = 5) to test the feasibility of the protocol. Recruitment to the pilot was limited due to an overarching view that participation would be onerous and that the questionnaires looked daunting. There was a general lack of interest in the study and self-reports of limited reading/writing ability among those approached to participate. There was a need to refine the study, e.g. improve the visual appearance of the questionnaires to make them more attractive to participants, offer flexibility and support to complete questionnaires, and to highlight benefits of participation via short video clips from an on-site study champion.

The results from the small pilot sample did enable an evaluation of the primary outcomes and the limitations of methodology. Data were successfully collected from four of five participants (one person failed to provide a second blood sample because they were off-site at the end of the pilot). Three of the five participants showed increased 25(OH)D levels between the first and second blood sample, and one returned a lower 25(OH)D reading at the end of the pilot. Assessment of the stage of change of the participants based on the questionnaire data showed that in terms of sun-safety they tended towards planning and actions stages, whereas in relation to healthy eating to increase Vitamin D levels they tended towards the (pre) contemplation or planning stages (possibly reflecting a lack of knowledge on this topic).

### Full study protocol

This paper presents the protocol for the full study intervention to promote sun-safe and healthy eating related behaviours (to increase Vitamin D) in construction workers. The study will use the TPB questionnaire [29] developed from formative work undertaken and the additional piloting. The intervention text messages will target two areas:

1. Promoting sun-safety behaviours (i.e. during the period when Vitamin D can be synthesised via UV in the UK from April–September) based on the previously identified beliefs, such as photo-ageing effects of the sun, increased skin cancer risk and reduced stigma about applying sunscreen at work.
2. Increasing knowledge and encouraging behaviours to improve Vitamin D intake and healthy eating habits (during the period when Vitamin D cannot be synthesised via UV from October to March), for example health recommendations or advice about food sources rich in Vitamin D.

We hypothesise that construction workers will report a change in their beliefs and sun-safe behaviours and healthier eating habits with respect to increasing their Vitamin D levels. During the study year, we expect to see a stabilising of the 25(OH)D profile rather than a peak in the summer and trough in the winter. This research will provide valuable information about the effectiveness of a technology-based intervention to promote sun-safety and healthy eating behaviours in outdoor construction workers.

## Methods and design

### Aim

The aim of the study is to investigate if a combination of short messages delivered to the smartphones of construction workers and a supportive sun-safe and healthy behaviour smartphone App, can influence workers to reduce their exposure to UV radiation in summer, and promote appropriate dietary changes in winter to increase their Vitamin D intake.

### Study design

The study is a randomised control crossover trial (RCCT), with approximately 60 participants from the construction industry, across 12 construction sites in the UK (six in central Scotland and six in southern England). Participants will be randomised to the intervention at site level and will complete both control and intervention conditions. Recruitment will be for three waves of data collection. Each wave will last for 21 days, with wave 1 in winter, wave 2 in summer and wave 3 in winter. This will enable Vitamin D level and seasonal UV light exposure to be profiled across the year.

### Study sample

#### Recruitment and eligibility

Approximately 60 construction workers will be recruited to the study from construction sites – via an onsite information session - in the UK (i.e. central Scotland and southern England). The criteria for eligibility will include male and female employees in the construction industry residing in the UK, both indoor and outdoor workers.

#### Sample size

Sample size calculations were based on change in 25(OH)D levels as the primary study outcome. Previous research studies have reported between individual variability in 25(OH)D levels, with standard deviations ranging from around 16–27 nmol/l (most individuals have 25(OH)D levels between about 30 and 90 nmol/l). Within-individual change in 25(OH)D level is likely to have a lower variability as it adjusts for some of the between individual variability. We assumed change in 25(OH)D has a standard deviation of 16 (at the low end of the reported range). Statistical power calculations showed that the study would have 80% power to detect differences of 10 nmol/l or more in change in 25(OH)D between the intervention group and the controls in each of the two seasons.

### Study conditions

#### Intervention

**Fig. 2:**
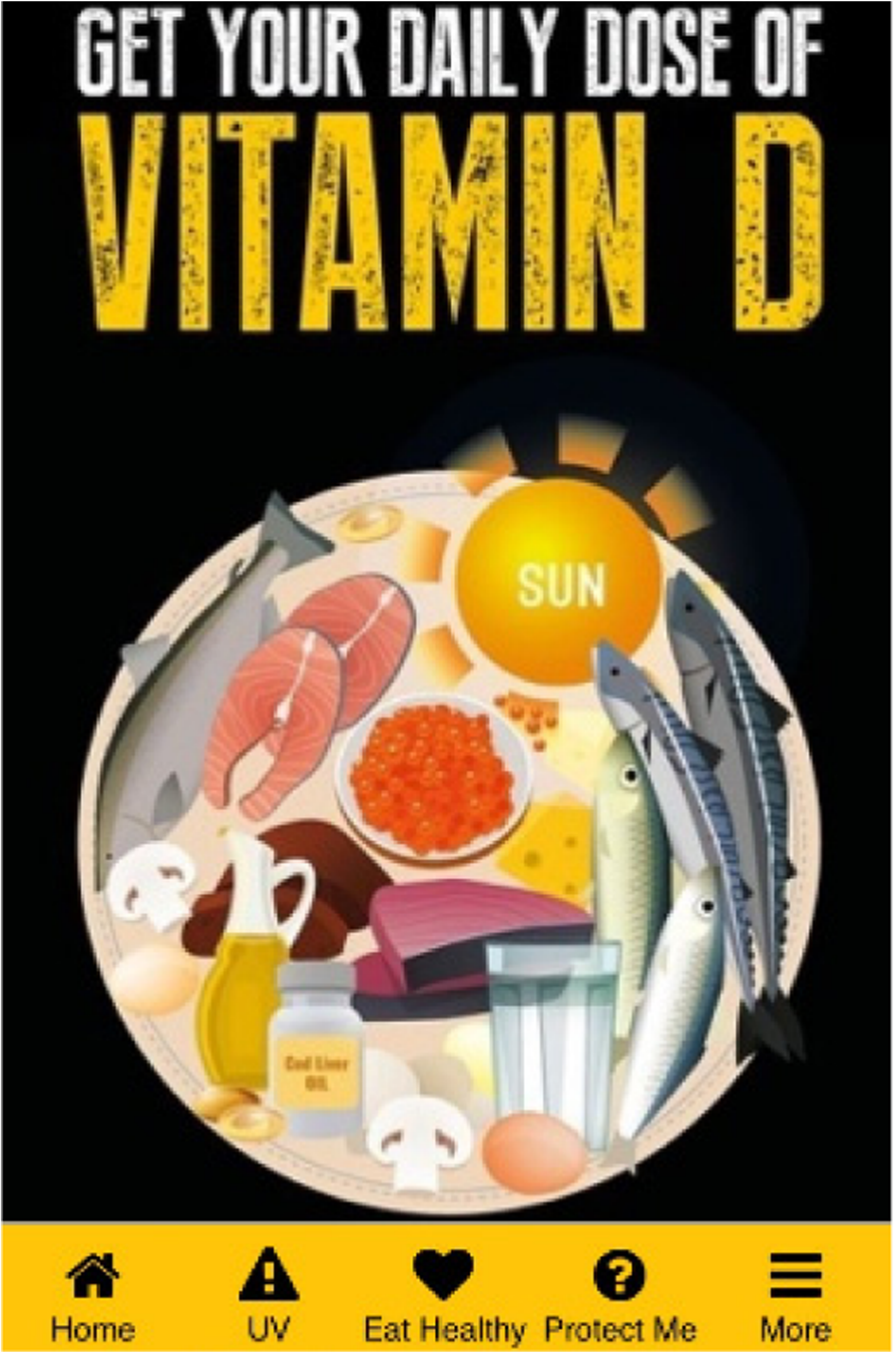
App page, Vitamin D. © IOSH, used with permission

**Fig. 3:**
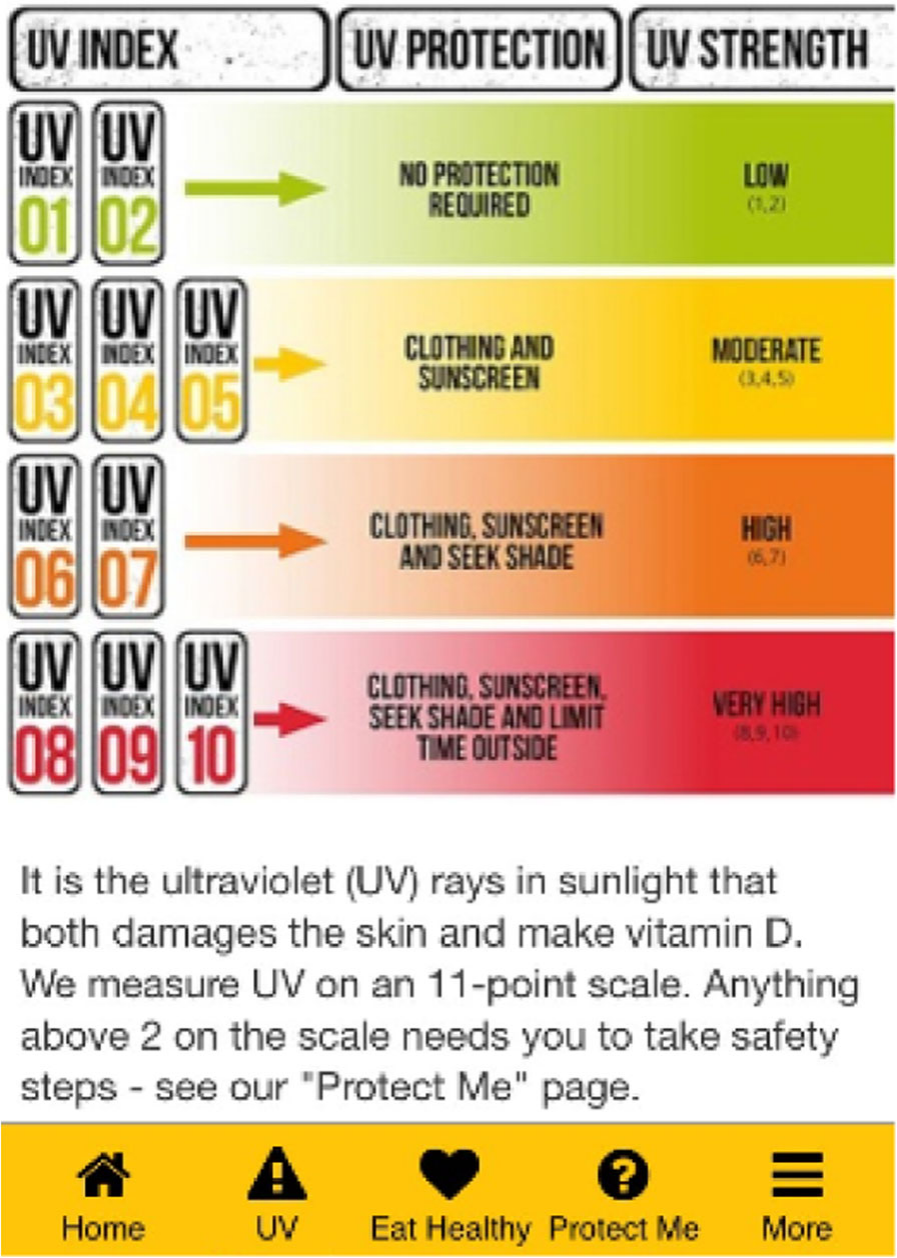
App page, UVI information, © IOSH, used with permission

**Fig. 4:**
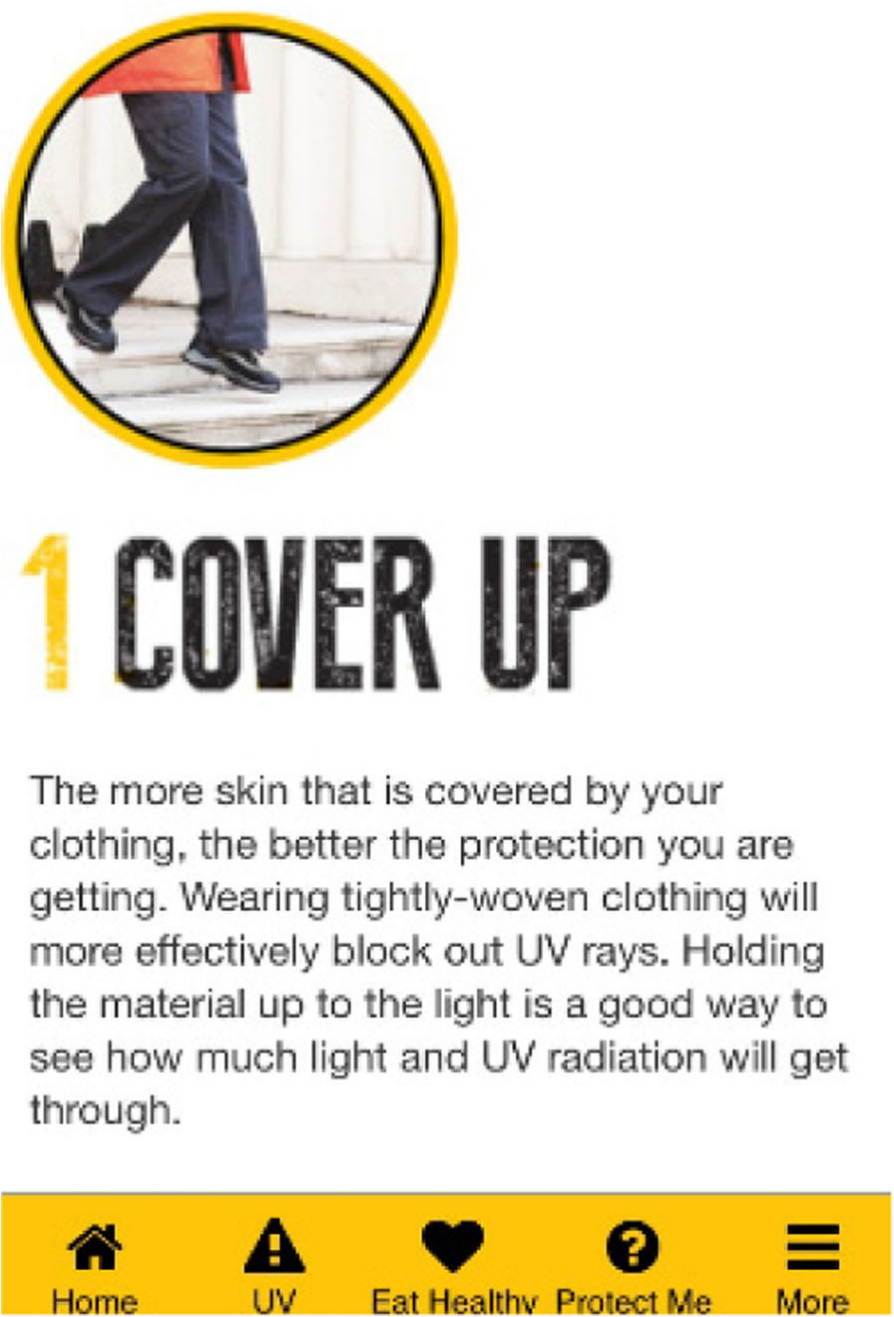
App page, ‘Protect me’, © IOSH, used with permission

The intervention will be delivered to the workers via a mobile phone short message service (SMS) and/or SMS containing hyperlinks/URL to relevant information. These messages have been tailored to reflect season, UV light exposure and/or strategies to increase Vitamin D intake in winter etc. Participants will receive daily messages in the morning, and supplementary messages dependent upon season/weather (e.g. if the UV Index is high across the intervention period, they may be prompted to protect themselves from the sun). The supportive App contains statistics about serious health outcomes linked to poor sun-safety behaviour, images of skin type and information on the need to protect fair/lighter coloured skin. Location driven UV radiation information and the actions to take at the time (e.g. the UV Index is high, cover up in the midday sun) will also be featured on the App, see Figs. 2, 3 and 4 [33]. Sources of Vitamin D (e.g. natural food or fortified products) and the benefits of dietary supplements will be highlighted. During the low UV intervention period participants will be issued with a Vitamin D dietary supplement (daily tablet 10 μg) and advised that they can take the tablet during the 3-week study period. The number of supplement consumed during the study will be recorded at the follow up visit.

#### Control

During the control period participants will not receive the intervention daily text messaging service or prompts to view the sun-safe and healthy behaviour App.

### Measurements

#### Primary outcomes

The main outcome measure is participant 25(OH)D level, collected using a simple non-invasive method. The 25(OH)D assay uses a liquid-liquid extraction method and tandem mass spectrometry (Waters TQD and Acquity UPLC) to measure 25-hydroxyvitamin D2 and D3. Deuterated internal standards are also measured for both 25-hydroxyvitamin D2 and D3 and only 150uL of serum/plasma is required for the analysis. Total 25-Hydroxyvitamin D (nmol/L) thresholds are defined as: Severe Deficiency < 15 nmol/L, Deficiency 15–30 nmol/L, Insufficiency 30.1–50 nmol/L, Adequate Status 50.1–220 nmol/L. Participants are provided with a home test kit using dried blood spot, which is easy to use. The kit contains a single-use lancet, alcohol wipe, blood-drop sampling card and sticking plaster. The procedure involves collecting four blood spots taken from the tip of their finger, on to the test card, via the ‘pin-prick’ method. The sample card and consent form are placed in a sealed envelope and posted to the lab for analysis. Participants will be notified that they are consenting to the blood sample being analysed by Pathology Department, City Hospital, Birmingham, (http://www.cityassays.org.uk) and additional consent ensures participants agree to the results being shared with the research team.

#### Secondary outcomes

During the period when Vitamin D can be synthesised via UV in the UK (April–September) participants will be issued with a UVB (230-320 nm erythemally-weighted) wearable sensor. This is a small dosimeter, manufactured by Scienterra Limited (New Zealand) designed to measure and record exposure to UVB radiation. UVB is the main cause of skin reddening and sunburn, which plays a key role in the development of skin cancer and a contributory role in tanning and photo-ageing [21]. The dosimeters will provide objective data on levels of UVB exposure received by study participants during the high UV period (April–September).

#### Questionnaires

At the start of the trial participants will complete a socio-demographic profile questionnaire (see Additional file 1)*.* This will record age, gender, skin type and occupational characteristics. All other assessments will be collected at the start and end of each 21-day data collection wave:

1. *Theory of Planned Behaviour*: The theoretical framework will be used to determine, at the start of the intervention, the intended behaviours over the 21 day study period, and again at the end of the study to identify if those behaviours were actually performed, (see Additional files 2 and 3) e.g. ‘*I will try to take sun-protective measures every time I work in the sun, over the next three weeks’, ‘I plan to increase my Vitamin D intake, every day, over the next three weeks’.* The TPB will also determine if beliefs about sun-safety or healthy eating change from the start to end of the study period, e.g. ‘*Performing sun-safety measures is something that I should do, in the next three weeks’, ‘Increasing my Vitamin D intake, every day, over the next three weeks will promote healthy bones’.*

(2) *Knowledge of sun-safe behaviour and Vitamin D:* Participants will be asked about their knowledge of sun-safety and/or level of agreement with statements about risks, (see Additional file 1) e.g. ‘*It is important to wear sunglasses to protect the eyes from the sun*?’ - Agree/disagree/don’t know. We will investigate their knowledge and experience of Vitamin D using the same format, e.g. ‘*I can get enough Vitamin D from the sun in the UK all year round*’, ‘*I’ve experienced Vitamin D deficiency*’, ‘*It’s important to eat oily fish in winter to boost Vitamin D intake*’.

(3) *Stages of change:* A matrix, published by Houdmont and Madgwick [23], will be used to determine participants’ stage of change with respect to their adoption of sun-safety measures, and the dietary measures that they currently take to increase their Vitamin D levels. Each participant will be asked to respond to a series of statements about their behaviours in relation to each of these measures (see Additional file 1) - ‘*I avoid/minimise working in sunlight in the middle of the day’; ‘* ‘*Check the UV index forecast for the day*’; ‘*Take a Vitamin D dietary supplement during the winter*’: ‘*Regularly eat Vitamin D rich foods, i.e. mushrooms, oily fish, eggs*’) by indicating which of the five stages of change is most appropriate to their current performance: 1) *I don’t do this and I’m not thinking about starting* (pre-contemplation), 2) *I don’t do this but I’m thinking about starting* (contemplation), 3) *I don’t do this, but I’m planning to start in the next month* (planning), 4) *I do this and began to do it in the last 12 months* (action), or 5) *I do this and have done so for more than a year* (maintenance).

### Data analysis

Examination of the effect of the intervention will be based on the primary response variable of change in 25(OH)D over the test period. The extent and direction of the change will be compared between groups in each of the high UV and low UV seasons using standard statistical methods, including comparison of mean levels of 25(OH)D change, comparison of proportion in each group whose levels increase or decrease; and within individual comparisons for intervention and control periods, which will control for differences between individuals in response to UV exposure. Secondary analyses will include additional comparisons of the level and direction of change in high and low UV periods in relation to geographic area of study (Scotland or England), different types of targeted intervention, and directly to the responses to the behavioural (i.e. TPB) and risk knowledge questionnaires.

## Discussion

This study trials a technology-based intervention for construction workers in the UK. The theory-driven design accounts for the psychological factors that may impact upon sun-safety and 25(OH)D levels among construction workers in the UK. If successful, results will demonstrate that a simple text messaging service could serve to promote sun-safety behaviours during the summer months and help to reduce the decline in Vitamin D levels during the winter period. The positive and negative effects of sun exposure at work are important health issues, particularly for outdoor workers. This work may provide an effective way of communicating sun-safety and other relevant health promotion messages. The methodological approach could be used to develop interventions to reduce the risk of skin cancer amongst construction workers in other parts of the world where these are issues for outdoor workers.

## Additional files

Additional file 1: Study questionnaire (Socio-demographics, Knowledge and Stages of Change). (DOCX 89 kb)

Additional file 2: Theory of Planned Behaviour, Sun-safety questionnaire. (DOCX 28 kb)

Additional file 3: Theory of Planned Behaviour, Vitamin D questionnaire. (DOCX 26 kb)

## Abbreviations

Acquity UPLC: Acquity Ultra-Performance Liquid Chromatograph
RCCT: Randomised control crossover trial
SMS: Short message service
TPB: Theory of Planned Behaviour
TQD: Waters tandem quadruple
UV: Ultra-violet
UVI: Ultra Violet Index
