# Supplementary material for: A randomised control crossover trial of a theory based intervention to improve sun-safe and healthy behaviours in construction workers: study protocol
Source: BMC Public Health. 2018 Feb 15;18:259. doi: 10.1186/s12889-018-5164-8 (PMC5815245; doi:10.1186/s12889-018-5164-8)
Supplement: Supplementary file 1 — Study questionnaire (Socio-demographics, Knowledge and Stages of Change). (DOCX 89 kb) [file 12889_2018_5164_MOESM1_ESM.docx]

**Part 1 YOURSELF**

**(Please tick or complete the response where indicated)**

| 1. **Gender** | 1. **Skin type** | | 1. **Location where you work** |
| --- | --- | --- | --- |
| Male  Female  Not specified | Very pale  Fair/pale  Fair/beige  Olive/light brown  Dark brown  Black | | South East  London  South West  East Anglia  Midlands  North East  North West  Scotland  Wales  Northern Ireland  Not specified |
| 1. **Age** | 1. **Ethnicity** | |  |
| ≤20  21-30  31-40  41-50  51-60  ≥61 | White UK/Irish  Mixed / Multiple ethnic groups  Asian / Asian British  Black / African / Caribbean / Black British  Other ethnic group………………………. | |  |
| 1. **Occupation characteristics** | | | |
| Apprentice  Asbestos remover  Banksman  Bricklayer  Civil engineer  Cladder  Customer care  Diamond driller  Electrician  Fence erector  Fireproofer  Fork lift driver  Ground worker  Health and safety professional  Insulator  Joiner  Labourer  Lift engineer  Mech Tech / Plumber  Painter  Plant fitter | | Plant/Machine operator  Plasterer  Plater  Rigger  Roofer  Scaffolder  Snr Construction Manager  Sign maker/fitter  Site engineer  Site foreman  Site office staff  Site manager  Site supervisor  Steel fixer  Storeman  Teacher / instructor  Tiler  Traffic management  Welder  Window fitter  Other, please specify ……………………… | |

**Part 2 KNOWLEDGE**

**(Please tick or complete the response where indicated)**

| 1. **Skin cancer experience** |
| --- |
| In the last 12 months how many times have you had a red sunburn that lasted a day or more?  0 1 2 3 4 5 more than 5 |
| Have you had skin cancer?  Yes No If so, when ………………………… |
| Have any members of your family or close friends had skin cancer?  Yes No |
| In the last month have you (please tick all that apply)………….  been on holiday in a sunny country, if so when and where……………..……………………..  used a tanning sunbed, if so how often…………………………………………………………..  regularly taken vitamin D supplements |
| 1. **Skin checking** |
| Have you ever had your skin checked for cancer by a health professional?  Yes No |
| In the last 12 months have you checked your whole body for moles or skin changes?  Yes No |
| In the last 12 months have you checked areas of your body regularly exposed to the sun for moles or skin changes?  Yes No |
| 1. **Training and sunscreen provision** |
| Is sunscreen supplied in your workplace?  Yes  No Don’t know |
| Have you ever had training on the risks of working in the sun?  Yes No |
| **Sun safety knowledge and attitudes** |
| I don’t need to wear sunscreen on a cloudy/overcast day during summer?  Agree Disagree Don’t know |
| It is important to wear sunglasses to protect the eyes from the sun?  Agree Disagree Don’t know |
| Sun exposure is the most important risk factor for skin cancer?  Agree Disagree Don’t know |
| If I apply factor 30 sun screen, then I need only apply it once per day?  Agree Disagree Don’t know |
| I like to have a suntan  Agree Disagree Don’t know |
| I think I am at risk of skin cancer  Agree Disagree Don’t know |
| Sun protection is important when working outside for less than one hour in the sun  Agree Disagree Don’t know |
| 1. **Vitamin D knowledge** |
| I can get enough vitamin D from the sun in Scotland all year round?  Yes No |
| Vitamin D is important for healthy eyesight and healthy skin?  Yes No |
| I’ve experienced vitamin D deficiency?  Yes No Don’t know |
| Have any members of your family or close friends had experienced vitamin D deficiency?  Yes No Don’t know |
| It’s important to eat oily fish in winter to boost vitamin D intake?  Yes No Don’t know |
| It’s important, in winter, to eat organic food and vegetables to boost vitamin D intake?  Yes No Don’t know |
| In winter, everyone in Scotland should take vitamin D supplements to keep healthy?  Yes No Don’t know |
| I get more vitamin D from natural food (i.e. veg, oily fish, eggs etc.) or supplements?  Natural food Dietary supplements |
| How long do I have to be in the sun to get enough vitamin D?  5 mins  10-15 mins on a sunny day  15-30 mins  1-3hours  All morning  All day |

**Part 3 Stages of change**

**(Please tick or complete the response where indicated)**

|  | I don’t do this  and I’m not  thinking about  starting  (*precontemplation*) | I don’t do this  but I’m thinking  about starting  (*contemplation*) | I don’t do this,  but I’m  planning to  start in the next  month  (*planning*) | I do this and  began to do it  in the last 12  months  (*action*) | I do this and  have done so  for more than a  year  (*maintenance*) |
| --- | --- | --- | --- | --- | --- |
| Avoid/minimise work  in summer sunlight in the  middle of the day |  |  |  |  |  |
| Swap jobs to  minimise the amount  of time working in  the sun |  |  |  |  |  |
| Use a shade/cover  when working in the  sun |  |  |  |  |  |
| Wear long sleeved,  loose fitting tops and  trousers on sunny days |  |  |  |  |  |
| Wear neck  protection with my safety  helmet in the sun |  |  |  |  |  |
| Wear sunglasses |  |  |  |  |  |
| Use sunscreen on sunny days |  |  |  |  |  |
| Drink plenty of water |  |  |  |  |  |
| Check the UV index  forecast for the day |  |  |  |  |  |
| Regularly check my skin  for moles or unusual  changes |  |  |  |  |  |

|  | I don’t do this  and I’m not  thinking about  starting  (*precontemplation*) | I don’t do this  but I’m thinking  about starting  (*contemplation*) | I don’t do this,  but I’m  planning to  start in the next  month  (*planning*) | I do this and  began to do it  in the last 12  months  (*action*) | I do this and  have done so  for more than a  year  (*maintenance*) |
| --- | --- | --- | --- | --- | --- |
| Take a Vitamin D dietary supplement during the winter |  |  |  |  |  |
| Have my Vitamin D levels checked by a health professional |  |  |  |  |  |
| Regularly eat Vitamin D rich foods, i.e. mushrooms, oily fish, eggs |  |  |  |  |  |
| Eat Vitamin D fortified cereals, e.g. Weetabix, ReadyBrek, Bran Flakes |  |  |  |  |  |
| Have Vitamin D fortified dairy products, e.g. Alpro soya milk, |  |  |  |  |  |
| Expose my skin to **only** 10-15 mins of sun to get my natural dose of Vitamin D |  |  |  |  |  |
