# Supplementary material for: A randomised control crossover trial of a theory based intervention to improve sun-safe and healthy behaviours in construction workers: study protocol
Source: BMC Public Health. 2018 Feb 15;18:259. doi: 10.1186/s12889-018-5164-8 (PMC5815245; doi:10.1186/s12889-018-5164-8)
Supplement: Supplementary file 2 — Theory of Planned Behaviour, Sun-safety questionnaire. (DOCX 28 kb) [file 12889_2018_5164_MOESM2_ESM.docx]

**Safe Working and the Sun**

**Background**

We are interested in the implications of the sun on the way you work. The amount of sun you are exposed to may have implications for your health. During Summer, many workers risk over-exposure to the sun. This questionnaire concerns things you may do to protect yourself from the sun.

The questionnaire should take about fifteen minutes to complete.

**Sun Protective Measures**

Potential sun protective measures include:

- using ‘high factor’ sunscreen, e.g., Sun Protection Factor (SPF) 30 or more
- working in the shade or indoors, if possible
- wearing a hat, with/out a neck shade
- wearing overalls, or
- long sleeved tops and trousers, not shorts, and/or
- using sunglasses.

**Your Behaviours and Opinions**

For the following statements, please…

1. Place your mark where it best reflects your opinion. For example, the following statement, ‘the weather in London in July is…’, was rated as ‘quite good’, by marking it as follows.

Good ________ : ________ : ________ : ________ : ________ : ________ : ________ Bad

1. Please respond to ALL statements, do not miss any out.
2. Please do not put more than one mark for each statement.
3. I expect sunscreen to be provided by my employer, every time I work in the sun, in the next three weeks.

Disagree _______ : _______ : _______ : _______ : _______ : _______ : _______ Agree

1. Decreasing my chances of getting skin cancer is…

Good _______ : _______ : _______ : _______ : _______ : _______ : _______ Bad

1. When it comes to taking sun-protective measures, how much do you want to do what your health and safety advisor thinks you should do?

Completely _______ : _______ : _______ : _______ : _______ : _______ : _______ Not at all

1. When it comes to taking sun-protective measures; how much do you want to do what your family thinks you should do?

Completely _______ : _______ : _______ : _______ : _______ : _______ : _______ Not at all

1. My employer’s support for working out of the sun, will make it…

Easy _______ : _______ : _______ : _______ : _______ : _______ : _______ Difficult

…for me to take sun-protective measures, in the next three weeks.

1. My family thinks that I…

Should not _______ : _______ : _______ : _______ : _______ : _______ : _______ Should

…take sun-protective measures every time I work in the sun, over the next three weeks.

1. My co-workers think that taking sun-protective measures, every time they work in the sun, over the next three weeks, is a good thing to do.

Agree _______ : _______ : _______ : _______ : _______ : _______ : _______ Disagree

1. When it comes to taking sun-protective measures, how much do you want to do what your doctor thinks you should do?

Completely _______ : _______ : _______ : _______ : _______ : _______ : _______ Not at all

1. My taking sun-protective measures every time I work in the sun, over the next three weeks will decrease my chances of getting skin cancer.

Unlikely _______ : _______ : _______ : _______ : _______ : _______ : _______ Likely

1. I intend to take sun-protective measures every time I work in the sun, over the next three weeks.

Unlikely _______ : _______ : _______ : _______ : _______ : _______ : _______ Likely

1. Most of my co-workers will take sun-protective measures, every time they work in the sun, over the next three weeks.

Agree _______ : _______ : _______ : _______ : _______ : _______ : _______ Disagree

1. It is mostly up to me whether I take sun-protective measures every time I work in the sun, over the next three weeks.

Agree _______ : _______ : _______ : _______ : _______ : _______ : _______ Disagree

1. My health and safety advisor thinks that I…

Should not _______ : _______ : _______ : _______ : _______ : _______ : _______ Should

…take sun-protective measures every time I work in the sun, over the next three weeks.

1. I think I should perform some sun protective measures, in the next three weeks.

Disagree _______ : _______ : _______ : _______ : _______ : _______ : _______ Agree

1. Many people like me take sun-protective measures every time they work in the sun.

Likely _______ : _______ : _______ : _______ : _______ : _______ : _______ Unlikely

1. I expect my employer to enable me to work in the shade or indoors, rather than in the sun, if needed, in the next three weeks.

Disagree _______ : _______ : _______ : _______ : _______ : _______ : _______ Agree

1. I plan to take sun-protective measures every time I work in the sun, over the next three weeks.

Disagree _______ : _______ : _______ : _______ : _______ : _______ : _______ Agree

1. Celebrities always seem to have a tan.

Agree _______ : _______ : _______ : _______ : _______ : _______ : _______ Disagree

1. Sunscreen provided by my employer will make it…

Easy _______ : _______ : _______ : _______ : _______ : _______ : _______ Difficult

…for me to take sun-protective measures, every time I work in the sun, in the next three weeks.

1. I will try to take sun-protective measures every time I work in the sun, over the next three weeks.

True _______ : _______ : _______ : _______ : _______ : _______ : _______ False

1. If I wanted to, I could take sun-protective measures every time I work in the sun, over the next three weeks.

True _______ : _______ : _______ : _______ : _______ : _______ : _______ False

1. Please estimate how often you have taken sun protective measures when working in the sun, over the last three weeks.

Never _______ : _______ : _______ : _______ : _______ : _______ : _______ Everyday

1. I can think of many celebrities who do **not** have a tan

Disagree _______ : _______ : _______ : _______ : _______ : _______ : _______ Agree

1. How many of your co-workers would think that taking sun-protective measures every time you work in the sun, over the next three weeks is a good thing to do? Please circle the appropriate response.

none

a few

nearly half

around half

more than half

almost all

all.

1. It is expected of me that I will take sun-protective measures every time I work in the sun, over the next three weeks.

Likely _______ : _______ : _______ : _______ : _______ : _______ : _______ Unlikely

1. Performing sun safety measures is something that I should do, in the next three weeks.

Agree _______ : _______ : _______ : _______ : _______ : _______ : _______ Disagree

1. Most people who are important to me take sun-protective measures every time they work in the sun.

True _______ : _______ : _______ : _______ : _______ : _______ : _______ False

1. How many times, in the last three weeks, have you taken sun protective measures when working in the sun? Please circle the appropriate response.

on every occasion

on almost every occasion

on most occasions

on around half the occasions

on nearly half the occasions

a few times

never.

1. The people in my life whose opinions I value…

Do not take _______ : _______ : _______ : _______ : _______ : _______ : _______ Take

…sun-protective measures every time they work in the sun.

1. For me to take sun-protective measures every time I work in the sun, over the next three weeks is…

Good _______ : _______ : _______ : _______ : _______ : _______ : _______ Bad

Harmful _______ : _______ : _______ : _______ : _______ : _______ : _______ Beneficial

Pleasant _______ : _______ : _______ : _______ : _______ : _______ : _______ Unpleasant

Enjoyable _______ : _______ : _______ : _______ : _______ : _______ : _______ Unenjoyable

1. On how many days in the last three weeks did you take sun protective measures, when working in the sun?

days.

1. My doctor thinks that I…

Should not _______ : _______ : _______ : _______ : _______ : _______ : _______ Should

…take sun-protective measures every time I work in the sun, over the next three weeks.

1. How many of your co-workers would take sun-protective measures every time they work in the sun, over the next three weeks? Please circle the appropriate response.

none

a few

nearly half

around half

more than half

almost all

all.
