# Supplementary material for: A randomised control crossover trial of a theory based intervention to improve sun-safe and healthy behaviours in construction workers: study protocol
Source: BMC Public Health. 2018 Feb 15;18:259. doi: 10.1186/s12889-018-5164-8 (PMC5815245; doi:10.1186/s12889-018-5164-8)
Supplement: Supplementary file 3 — Theory of Planned Behaviour, Vitamin D questionnaire. (DOCX 26 kb) [file 12889_2018_5164_MOESM3_ESM.docx]

**Safe Working and the Sun**

**Background**

We are interested in the implications of the sun on the way you work. The amount of sun you are exposed to may have implications for your health. During Winter, many workers are unable to get the Vitamin D they require from the Sun. This questionnaire concerns your Vitamin D intake.

The questionnaire should take about fifteen minutes to complete.

**Vitamin D Intake**

Potential sources of Vitamin D include:

- taking dietary supplements, e.g., tablets
- eating oily fish
- spending 15 minutes in the sun
- eating eggs or cheese, or
- taking fortified produce, e.g., milk or cereal.

**Your Behaviours and Opinions**

For the following statements, please…

1. Place your mark where it best reflects your opinion. For example, the following statement, ‘the weather in London in July is…’, was rated as ‘quite good’, by marking it as follows.

Good ________ : ________ : ________ : ________ : ________ : ________ : ________ Bad

1. Please respond to ALL statements, do not miss any out.
2. Please do not put more than one mark for each statement.
3. I plan to increase my Vitamin D intake, every day, over the next three weeks.

Disagree _______ : _______ : _______ : _______ : _______ : _______ : _______ Agree

1. If I wanted to, I could take measures to increase my Vitamin D intake daily, over the next three weeks.

True _______ : _______ : _______ : _______ : _______ : _______ : _______ False

1. When it comes to taking measures to increase your Vitamin D intake; how much do you want to do what your Health Care Professional thinks you should do?

Completely _______ : _______ : _______ : _______ : _______ : _______ : _______ Not at all

1. For me to increase my Vitamin D intake, every day, over the next three weeks is…

Good _______ : _______ : _______ : _______ : _______ : _______ : _______ Bad

Harmful _______ : _______ : _______ : _______ : _______ : _______ : _______ Beneficial

Worthless _______ : _______ : _______ : _______ : _______ : _______ : _______ Valuable

1. Health Care Professionals think that I…

Should not _______ : _______ : _______ : _______ : _______ : _______ : _______ Should

…take measures to increase my Vitamin D intake daily.

1. Most people who are important to me take measures to increase their Vitamin D intake daily.

True _______ : _______ : _______ : _______ : _______ : _______ : _______ False

1. Making my bones healthier is…

Good _______ : _______ : _______ : _______ : _______ : _______ : _______ Bad

1. My friends/family think that I…

Should not _______ : _______ : _______ : _______ : _______ : _______ : _______ Should

…take measures to increase my Vitamin D intake daily.

1. Increasing my Vitamin D intake, every day, over the next three weeks will promote healthy bones.

Unlikely _______ : _______ : _______ : _______ : _______ : _______ : _______ Likely

1. I think the cost of increasing my Vitamin D will make it…

Easy _______ : _______ : _______ : _______ : _______ : _______ : _______ Difficult

…for me to take measures to increase my Vitamin D intake daily, over the next three weeks.

1. The people in my life whose opinions I value…

Disapprove _______ : _______ : _______ : _______ : _______ : _______ : _______ Approve

…of me take measures to increase my Vitamin D intake, every day, over the next three weeks.

1. I intend to increase my Vitamin D intake, every day, over the next three weeks.

Unlikely _______ : _______ : _______ : _______ : _______ : _______ : _______ Likely

1. Taking measures to increase my Vitamin D intake daily, over the next three weeks will be costly for me.

Disagree _______ : _______ : _______ : _______ : _______ : _______ : _______ Agree

1. When it comes to taking measures to increase your Vitamin D intake; how much do you want to do what your friends/family think you should do?

Completely _______ : _______ : _______ : _______ : _______ : _______ : _______ Not at all

1. Access to sources of Vitamin D while at work, will make it…

Easy _______ : _______ : _______ : _______ : _______ : _______ : _______ Difficult

…for me to take measures to increase my Vitamin D intake daily, over the next three weeks.

1. It is mostly up to me whether I take measures to increase my Vitamin D intake daily, over the next three weeks.

Agree _______ : _______ : _______ : _______ : _______ : _______ : _______ Disagree

1. How much control do you believe you have over taking measures to increase your Vitamin D intake daily, over the next three weeks.

None _______ : _______ : _______ : _______ : _______ : _______ : _______ Complete

1. It will be difficult to get sources of Vitamin D whilst at work.

Disagree _______ : _______ : _______ : _______ : _______ : _______ : _______ Agree

1. Most people who are important to me think that I…

Should not _______ : _______ : _______ : _______ : _______ : _______ : _______ Should

…take measures to increase my Vitamin D intake, every day, over the next three weeks.

1. I will try to increase my Vitamin D intake, every day, over the next three weeks.

True _______ : _______ : _______ : _______ : _______ : _______ : _______ False
